# A global view on the effect of water uptake on aerosol particle light scattering

**DOI:** 10.1038/s41597-019-0158-7

**Published:** 2019-08-22

**Authors:** María A. Burgos, Elisabeth Andrews, Gloria Titos, Lucas Alados-Arboledas, Urs Baltensperger, Derek Day, Anne Jefferson, Nikos Kalivitis, Nikos Mihalopoulos, James Sherman, Junying Sun, Ernest Weingartner, Paul Zieger

**Affiliations:** 1 Department of Environmental Science and Analytical Chemistry, Stockholm University, SE-10691 Stockholm, Sweden; 2 Bolin Centre for Climate Research, SE-10691 Stockholm, Sweden; 3 Cooperative Institute for Research in Environmental Studies, University of Colorado, Boulder, USA; 4 Andalusian Institute for Earth System Research, University of Granada, Granada, Spain; 5 Laboratory of Atmospheric Chemistry, Paul Scherrer Institute, Villigen, Switzerland; 6 Cooperative Institute for Research in the Atmosphere, Colorado State University, Fort Collins, USA; 7 Earth Systems Research Laboratory, National Oceanic and Atmospheric Administration, Boulder, Colorado USA; 8 Environmental Chemical Processes Laboratory, Department of Chemistry, University of Crete, Heraklion, Greece; 9 Department of Physics and Astronomy, Appalachian State University, Boone, USA; 10 Key Laboratory for Atmospheric Chemistry, Institute of Atmospheric Composition, Chinese Academy of Meteorological Sciences, Beijing, China; 11 Institute for Sensing and Electronics, University of Applied Sciences, Windisch, Switzerland

**Keywords:** Atmospheric science, Environmental sciences

## Abstract

A reference dataset of multi-wavelength particle light scattering and hemispheric backscattering coefficients for different relative humidities (RH) between RH = 30 and 95% and wavelengths between *λ* = 450 nm and 700 nm is described in this work. Tandem-humidified nephelometer measurements from 26 ground-based sites around the globe, covering multiple aerosol types, have been re-analysed and harmonized into a single dataset. The dataset includes multi-annual measurements from long-term monitoring sites as well as short-term field campaign data. The result is a unique collection of RH-dependent aerosol light scattering properties, presented as a function of size cut. This dataset is important for climate and atmospheric model-measurement inter-comparisons, as a means to improve model performance, and may be useful for satellite and remote sensing evaluation using surface-based, *in*-*situ* measurements.

## Abstract (summary)

**Table:** 

Design Type(s)	spectral data collection and processing objective • data integration objective • time series design
Measurement Type(s)	light scattering
Technology Type(s)	Nephelometry
Factor Type(s)	geographic location • instrument • Environment • temporal_interval
Sample Characteristic(s)	United States of America • climate system • Canada • The Netherlands • Greece • Germany • Portuguese Republic • South Korea • China • United Kingdom • Finland • Switzerland • Maldives Archipelago • Brazil • Republic of Ireland • Niger • India • Kingdom of Spain • Kingdom of Norway

Machine-accessible metadata file describing the reported data (ISA-Tab format)

## Background & Summary

Aerosol particles perturb the Earth’s radiation budget directly by scattering and absorbing solar radiation and, indirectly, through their role as cloud condensation nuclei. Particle size and refractive index vary with particle water content; making aerosol optical properties highly sensitive to changes in the ambient relative humidity (RH)^1–3^. This humidity dependence is one of the most uncertain parameters affecting aerosol direct radiative forcing^4–6^. Moreover, the aerosol hygroscopicity strongly influences cloud condensation nuclei (CCN) activity and, thus, cloud formation and cloud optical properties^7–9^. An accurate assessment of aerosol scattering enhancement with RH is necessary for validation of remote sensing retrievals^10–13^ and model evaluation^14–17^.

Long-term monitoring sites typically attempt to make their aerosol measurements at low RH (<40%), as recommended by the World Meteorological Organization/Global Atmosphere Watch (WMO/GAW) protocols for *in*-*situ* aerosol measurements^18^. While aerosol measurements at low RH are unlikely to be representative of many atmospheric conditions, operating at low RH makes the observations comparable across otherwise disparate sites and allows research to focus on the inherent optical characteristics of the dry aerosol without the confounding effect of aerosol water. However, to evaluate the climate effect of aerosol particles, knowledge about the RH-dependency of aerosol optical properties (particularly the particle light scattering coefficient) is needed.

The scattering enhancement factor, *f*(RH, *λ*), describes the change in particle light scattering coefficient *σ*_sp_(*λ*) as a function of RH:

1 $$f({\rm{R}}{\rm{H}},{\rm{\lambda }})=\frac{{\sigma }_{{\rm{s}}{\rm{p}}}({\rm{R}}{\rm{H}},{\rm{\lambda }})}{{\sigma }_{{\rm{s}}{\rm{p}}}({{\rm{R}}{\rm{H}}}_{{\rm{d}}{\rm{r}}{\rm{y}}},{\rm{\lambda }})},$$

where *λ* denotes the wavelength and RH_dry_ the dry relative humidity. The backscattering enhancement factor, *f*_b_(RH), is defined analogously, by replacing σ_sp_ with the backscattering coefficient σ_bsp_. For brevity, the explicit dependence on wavelength will be omitted from here on and the particle light scattering coefficient and enhancement factor will be written as *σ*_sp_ (RH) and *f*(RH), respectively.

A nephelometer is commonly used to measure *σ*_sp_ and, in conjunction with a humidifier system, allows determination of *σ*_sp_(RH) and *f*(RH), using the dry *σ*_sp_ as a reference. Tandem humidified nephelometer systems were developed in the early 1960s^19^, and have been continuously improved^20–22^, resulting in the most current versions of humidograph systems^23–26^. Titos *et al*.^27^ provide a comprehensive review of experimental designs of commonly used tandem humidified nephelometer systems.

Scattering enhancement factors depend on aerosol type and are described in previous studies (see e.g., Zieger *et al.*^16^, and Titos *et al*.^27^ and references therein). Small differences in the experimental design, operation procedures, calibration and data processing can lead to significant differences in the *f*(RH) values retrieved^16,27^. For example, dry conditions are not always defined the same way and some sites, especially sites in marine and tropical regions, have difficulties in maintaining the desired dry RH conditions.

Here, we present a re-processed, harmonized and quality-assured dataset of dry and RH-dependent particle total and back scattering coefficients (*σ*_sp_, *σ*_bsp_) from 26 sites across the globe, including measurements from nine sites which have not been previously reported. Using both dry and humidified values of *σ*_sp_ allows calculation of *f*(RH) according to Eq. 1. Since *f*(RH) or *σ*_sp_(RH) measurements are not routinely performed at most long-term surface monitoring stations, campaign-based data have been also included in this dataset to increase the number of sites and regions studied.

The general instrumental set-up and location-related specifics are described in the methods section as well as uncertainty estimates of *f*(RH). Individual files are provided as ASCII files for each station, size cut, year and data level. The individual files are available through the EBAS data portal and as joint package on the ACTRIS Data Centre (see links and DOI below).

This harmonized multi-site dataset of RH-dependent aerosol particle light scattering coefficients facilitates climatological studies of *f*(RH) as well as investigation of particle deliquescence. Two of the dataset’s primary applications are interpretation of satellite and ground-based remote sensing retrievals and evaluation of global climate models to reduce hygroscopicity-related uncertainty in radiative forcing. The dataset can also be used to study relationships between hygroscopicity and co-located and contemporaneous aerosol properties (e.g., chemical composition, single scattering albedo, etc.), potentially resulting in simplified and computationally efficient parametrisations similar to previous *f*(RH) proxy studies^28,29^.

## Methods

### Sites

This study utilises data from 26 sites across the globe, operating both on a campaign basis and as part of long-term monitoring efforts. Table 1 gives an overview of the sites and defines the acronyms used throughout the manuscript.

**Table 1:** General site information.

Station ID	Station name, Country	Instrument design	Lat.	Lon.	Alt. (m a.s.l.)	Site Type	Period (mm.yy)	Reference
APP	Appalachian State, USA	NOAA	36.2	−81.7	1100	Pol. Rural	05.12–12.16	—
BRW	North Slope of Alaska, USA	NOAA	71.3	−156.6	8	Arctic	08.06–10.13	—
CBG	Chebogue Point, Canada	NOAA	43.8	−66.1	5	Marine	07.04–08.04	Ervens *et al*.^9^
CES	Cabauw, Netherlands	PSI	52	4.9	60	Rural	06.09–10.09	Zieger *et al*.^12^
FIK	Finokalia, Greece	U. Crete	35.3	25.7	250	Marine	01.12–12.12	—
FKB	Black Forest, Germany	NOAA	48.5	8.4	511	Rural	03.07–12.07	Fierz-Schmidhauser *et al*.^23^
GRW	Graciosa, Portugal	NOAA	39.1	−28	15	Marine	04.09–12.10	—
GSN	Gosan, S. Korea	NOAA	33.28	126.2	72	Pol. Marine	04.01–12.01	Doherty *et al*.^38^
HFE	Shouxian, China	NOAA	32.56	116.8	23	Urban	05.08–12.08	Liu *et al*.^40^
HLM	Holme Moss, UK	NOAA	53.5	−1.9	525	Pol. Rural	11.06–12.06	—
HYY	Hyytiälä, Finland	PSI	61.9	24.3	180	Rural	05.13–08.13	Zieger *et al*.^26^
JFJ	Jungfraujoch, Switzerland	PSI	46.6	8	3580	Mountain	06.10–02.11	Zieger *et al*.^13^, Bukowiecki *et al*.^44^
KCO	Kaashidhoo Climate Observatory, R. Maldives	NOAA	4.9	73.5	1	Marine	02.99–03.99	Eldering *et al*.^47^
LAN	Lin’an, China	NOAA	30.3	119.7	138	Pol. Rural	03.13–03.13	Zhang *et al*.^49^
MAO	Manacapuru, Brazil	NOAA	−2.6	−60.2	50	Urban	08.14–04.15	—
MEL	Melpitz, Germany	PSI	51.4	12.9	86	Pol. Rural	02.09–03.09	Zieger *et al*.^51^
MHD	Mace Head, Ireland	PSI	53.3	−9.9	5	Marine	01.09–02.09	Fierz-Schmidhauser *et al*.^52^
NIM	Niamey, Niger	NOAA	13.5	2.2	205	Desert	01.06–12.06	—
PGH	Nainital, India	NOAA	29.4	79.5	1951	Urban	06.11–12.11	Gogoi *et al*.^55^, Dumka *et al*.^54^
PVC	Cape Cod, USA	NOAA	42.1	−70.2	1	Marine	07.12–06.13	Titos *et al*.^29^
PYE	Point Reyes, USA	NOAA	38.1	−123	5	Marine	03.05–09.05	—
SGP	Southern Great Plains, USA	NOAA	36.6	−97.5	315	Pol. Rural	12.98–12.16	Jefferson *et al*.^57^
THD	Trinidad Head, USA	NOAA	41.1	−124.2	107	Marine	04.02–03.06	—
UGR	Granada, Spain	U. Granada	37.2	−3.6	680	Urban	01.14–04.16	Titos *et al*.^25^
YOS	Yosemite, USA	US NPS	37.7	−119.7	1615	Pol. Rural	07.02–09.02	Malm *et al*.^60^
ZEP	Zeppelin, Norway	PSI	78.9	11.9	475	Arctic	07.08–10.08	Zieger *et al*.^61^

The site type refers to the predominant aerosol type. References listed refer to published *f*(RH) data from the site; further site references are given in the text.

A majority of the data come from the USA Department of Energy Atmospheric Radiation Measurements (DOE/ARM) deployments either at their long-term atmospheric observatory (SGP) or via the ARM Mobile Facility (AMF) campaigns (FKB, GRW, HFE, MAO, NIM, PGH, PVC, and PYE). Another large subset of the data was obtained during field campaigns in Europe (6 sites: CES, HYY, JFJ, MEL, MHD, and ZEP) performed by a research group from the Paul Scherrer Institute (PSI) in Switzerland. A detailed comparison of the PSI sites, recommendations for instrument operation and closure studies can be found in Zieger *et al*.^16^. More data was obtained from long-term monitoring sites in the USA National Oceanic and Atmospheric Administration Federated Aerosol Network (NOAA-FAN) (4 sites: APP, BRW (supported by DOE), THD and UGR) and shorter field campaign deployments by the NOAA-FAN research group (4 sites: CBG, GSN, HLM, and KCO). A few additional institutes like the University of Crete, the Chinese Academy of Meteorological Sciences (CAMS), and the USA National Park Service’s Interagency Monitoring of Protected Visual Environments (IMPROVE) program have also provided data from their deployments of tandem nephelometer systems (FIK, LAN and YOS, respectively; see Table 1 for references).

**Fig. 1:**
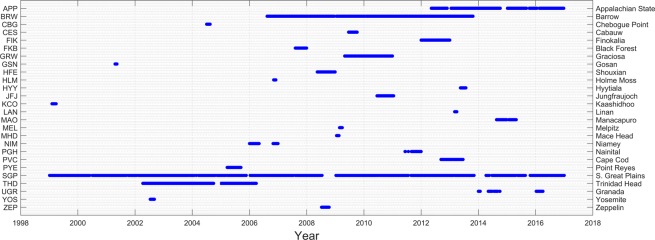
Data availability for all analysed sites.

This study presents, for the first time, the *f*(RH) results from the following sites: APP, BRW, FIK, GRW, HLM, MAO, NIM, PYE, and THD. Five of the sites (APP, BRW, GRW, SGP and THD) provide more than one year of continuous hygroscopicity measurements, enabling the investigation of annual cycles and climatologies in *f*(RH). Figure 1 shows data coverage for each site. More information about the measurement stations is provided below. The air sampling infrastructure at all DOE/ARM and NOAA sites utilizes the inlet system developed by NOAA/ESRL and follows GAW aerosol sampling protocols^18,22^. Other sites have individual characteristics which are briefly described below and in more detail in the provided references.

#### Appalachian State (APP), USA

The Appalachian Atmospheric Interdisciplinary Research Facility (APP) is situated at the highest point on the Appalachian State University campus (1080 m a.s.l.), in the heavily forested southern Appalachian Mountain region of North Carolina in the south-eastern USA. Although there are no major local aerosol sources (other from commuter and tourist traffic) the aerosol inlet is located 34 m above ground to minimize sampling of local sources. Secondary organic aerosol (largely isoprene-derived in summer) and sulphates dominate the sub-micron aerosol mass sampled at APP, along with a biomass burning influence during non-summer months^30^. More details about the site and the temporal variability of light scattering coefficient can be found in Sherman *et al*.^31^.

#### Barrow (BRW), USA

The Barrow facility is a coastal Arctic site in northern Alaska operated by NOAA/ESRL. The station is surrounded by flat tundra, large lagoons and lakes, and is approximately 1 km from the Arctic Ocean. The predominant wind direction is from east-north-east from the Beaufort Sea with minimal anthropogenic pollution. Generally, the station can be described as having an Arctic maritime climate. A description of the site as well as statistics and temporal variability of light scattering coefficient (among other optical properties) can be found in Delene *et al*.^32^.

#### Chebogue Point (CBG), Canada

A short-term, ground-based field site was established as part of the International Consortium for Atmospheric Research on Transport and Transformation (ICARTT) at Chebogue Point^33^. This coastal site was located at the south-west tip of Nova Scotia, Canada, 9 km south-south-west of the small town of Yarmouth. The Maine/New Brunswick coastline lies 130 km to the north-west across the Gulf of Maine. The cities of Boston and New York are 430 km and 730 km, respectively, to the south-west. Further site-specific information can be found in Ervens *et al*.^9^.

#### Cabauw (CES), Netherlands

The Cabauw Experimental Site for Atmospheric Research (CESAR^34^) is located about 40 km from the North Sea at 0.7 m below sea level, while air is sampled at around 60 m a.s.l. The station’s environment is typical for north-west Europe and can be described as background rural and maritime, depending on the wind direction and air mass influences. Further site information and previous results of *f*(RH) measurements and their link to hygroscopicity and remote-sensing data can be found in Zieger *et al*.^12^.

#### Finokalia (FIK), Greece

The remote coastal site of Finokalia, representative of the Eastern Mediterranean area, is located in the north-eastern coast of the island of Crete at the top of a hill at around 250 m a.s.l. While FIK is primarily a remote marine location, long-range transport episodes from Athens, central Europe, Asia, and North Africa can strongly affect the site. Aerosol measurements are conducted in a dedicated building at the station equipped with various aerosol inlets which sample at 4 m above ground level. More site-specific details can be found in Kalivitis *et al*.^35^.

#### Black Forest (FKB), Germany

This dataset was obtained within the context of the Convective and Orographic Induced Precipitation Study (COPS) field campaign in Heselbach, Germany. The site is located in a low mountain valley of the Black Forest surrounded by agricultural activity and is downwind of Stuttgart. Thus, the aerosol measured at FKB are primarily representative of rural continental air with occasional incursions from urban sources. The site has a typical mid-latitude moderate climate. Measurements reported here come from a deployment of the DOE/ARM Mobile Facility^36^. Fierz-Schmidhauser *et al*.^23^ presents the comparison between data measured by the NOAA and PSI systems, while in this study only data from the NOAA system has been analysed.

#### Graciosa (GRW), Portugal

The DOE/ARM Mobile Facility was deployed on the island of Graciosa to support the campaign Clouds, Aerosol and Precipitation in the Marine Boundary Layer (CAP-MBL). Graciosa is situated within the Azores archipelago in the eastern Atlantic Ocean. This marine site lies in the boundary between the subtropics and the mid-latitudes and experiences a wide range of meteorological conditions, ranging from undisturbed trade wind flow to cyclonic systems or extensive low-level stratus clouds. While the site is dominated by clean marine air masses, it can experience periodic episodes of polluted air masses from North America and Europe, and dust from the Saharan desert. More details on the GRW site can be found in Wood *et al*.^37^.

#### Gosan (GSN), South Korea

The Gosan supersite on Jeju Island, off the southern tip of South Korea, measured aerosol optical properties during the ACE-Asia campaign. Wintertime and spring flow is predominantly out of the north-west, carrying dust from the Loess regions, sea salt and pollution from coastal China. Local pollution includes burning and night time fishing vessels. The station is at the top of a 72 m a.s.l. cliff and the inlet at a height of 10 m above the ground. A site description and study of aerosol optical properties measured at Gosan are provided in Doherty *et al*.^38^.

#### Shouxian (HFE), China

The ARM-China campaign deployed the DOE/ARM Mobile Facility to Shouxian, in the Anhui province of China, located around 500 km west of Shanghai. The site lies within the rural region on Jiang-Huai between the Huai and Yangtze rivers. The site is located at the edge of a rural town and is largely surrounded by farmland. The weather is influenced by the East Asian monsoon system and the site is characterized by mixed agricultural, pollution and dust aerosol from road and building construction in nearby Nanjing. More details about HFE aerosol sampling system can be found in Jefferson *et al*.^39^ and previous results in Liu *et al*.^40^.

#### Holme Moss (HLM), UK

The Holme Moss site is located in Yorkshire in north-western England, approximately 30 km to the north-east of the city of Manchester and is characterized as a polluted rural site^41^. Aerosol measurements were made at HLM as part of a joint field campaign of the NOAA-FAN and University of Manchester research groups. The site is described in more detail in Liu *et al*.^41^.

#### Hyytiälä (HYY), Finland

The station SMEAR II is located in Hyytiälä, southern Finland^42,43^. This is an established long-term site surrounded by dense forests. The largest nearby city is Tampere, at around 60 km south-west. In this study the measurements from the campaign carried out as part of the EU-FP7 project PEGASOS are analysed. Details about instrument and campaign settings, previous results, including comparisons to aerosol mass spectrometer and airborne profile measurements, can be found in Zieger *et al*.^26^.

#### Jungfraujoch (JFJ), Switzerland

During the Cloud and Aerosol Characterization Experiments (CLACE) campaign measurements were performed in the Jungfraujoch research station^44^. Due to its high altitude (3580 m a.s.l.), JFJ is situated in the free troposphere most of the time. Nevertheless, thermal convection transports air from the planetary boundary layer to the site (especially during summer) and also long-range transport events such as African desert dust intrusions^13^ or volcanic ash from Iceland can be observed^45^. More information on the humidified nephelometer measurements at JFJ and the site in general can be found in Fierz-Schmidhauser *et al*.^46^, Zieger *et al*.^13^ and Bukowiecki *et al*.^44^.

#### Kaashidhoo Climate Observatory (KCO), R. Maldives

Situated on the remote Kaashidhoo Island in the Republic of the Maldives, the KCO site is not affected by local activity. During the dry monsoon season (December–April) airflow is from the Indian subcontinent. During the first half of the campaign (Feb 14–Mar 19, 1999) the air masses sampled originated from the Bay of Bengal and Calcutta region, while from Mar 10–Mar 28 winds were from the Arabian Sea. The measurements described here took place as part of the Indian Ocean Experiment (INDOEX) campaign and details can be found in Eldering *et al*.^47^ and Ramanathan *et al*.^48^.

#### Lin’an (LAN), China

The Lin’an Regional Atmosphere Background Station is located in the center of the Yangtze River Delta, China. It is approximately 11 km north of the city of Lin’an, ~50 km west of Hangzhou, and ~210 km south-west of Shanghai. The Lin’an station is on the top of a small hill, in an area primarily covered by bamboo forests and rice paddies, and represents the polluted background conditions of the Yangtze River Delta. Previous results of the relative humidity dependence of aerosol light-scattering for LAN have been presented in Zhang *et al*.^49^.

#### Manacapuru (MAO), Brazil

During the GOAMAZON campaign (‘Green Ocean Amazon’, January 2014 to November 2015) the DOE/ARM Mobile Facility was located downwind of the city of Manaus, near Manacapuru (Brazil). This site located near the Amazon rain forest and experiences pollution originating from Manaus, an industrial and urban region with soot and high-sulphur oil emissions. Biomass-burning emissions dominate particle emissions for much of the dry season. More details about the GOAMAZON campaign and instrumentation can be found in Martin *et al*.^50^.

#### Melpitz (MEL), Germany

The central European research station in Melpitz, Germany, is operated by the Leibniz Institute for Tropospheric Research (TROPOS). The site is surrounded by agricultural pastures, forests and small villages. The largest cities close by are Leipzig located at about 50 km south-west and Torgau located about 5 km north east of the site. Results related to humidified nephelometer measurements from this site are presented in Zieger *et al*.^51^.

#### Mace Head (MHD), Ireland

The Mace Head Atmospheric Research Station, on the west coast of Ireland, is located on a peninsula surrounded by coastline (70 to 120 m from the shoreline). Marine and clean air conditions were the dominant air mass types sampled at MHD, along with occasional non-marine and polluted air masses. Previous results about the hygroscopicity measurements can be found in Fierz-Schmidhauser *et al*.^52^.

#### Niamey (NIM), Niger

The DOE/ARM Mobile Facility deployment in Niamey during 2006 was associated with two large international campaigns: the African Monsoon Multidisciplinary Analysis (AMMA) and the Geostationary Earth Radiation Budget (GERB) experiment. Niamey, the capital of Niger, is located in the south-east region of the country, next to the Niger River. Due to both its location and the local meteorology the region experiences episodes of mineral dust (from the Sahara) and biomass burning aerosols in the dry season and deep tropical convection in the wet season. The measurement site was located near the Niamey Airport, close to runway traffic and jet exhaust plumes. A detailed description of the site and instrument set-up is described in Miller *et al*.^53^.

#### Nainital (PGH), India

Nainital is located in the foothills of the central Himalayas at an altitude of 1958 m a.s.l. The aerosol measurements were performed at the Aryabhatta Research Institute for Observational Sciences observatory at Manora Peak during the Ganges Valley Aerosol Experiment (GVAX). Nainital is impacted by both local and transported aerosols plumes. At specific time periods (winter time, early morning and late evening) the growth of the planetary boundary layer plays a major role in transporting aerosols from the valleys to the site, producing significant perturbations in aerosol properties. Measurements reported here come from a deployment of the DOE/ARM Mobile Facility during a 9-month campaign. Results of the *f*(RH) measurements for PGH have been previously presented in Dumka *et al*.^54^ and Gogoi *et al*.^55^.

#### Cape Cod (PVC), USA

The measurements at Cape Cod were conducted by the DOE/ARM during the Two-Column Aerosol Project (TCAP) deployment at Cape Cod, Massachusetts. Cape Cod is a peninsula jutting out into the Atlantic Ocean in the easternmost portion of the state of Massachusetts, in the north-eastern USA. The deployment was located in the north-eastern part of the cape, inside the Cape Cod National Seashore, and relatively close to large urban agglomerations such as Providence and Boston. Due to its location, the site is subject to both clean maritime and polluted conditions. Titos *et al*.^29^ present previous aerosol hygroscopicity results for this site.

#### Point Reyes (PYE), USA

This site is located at Point Reyes National Seashore, 70 km north of San Francisco, California, 1.6 km off the Pacific Ocean. The measurements were made as part of the DOE/ARM test deployment of their first Mobile Facility in 2005 and contributed to the MaSE (Marine Stratus Experiment) campaign. While the site is primarily a clean marine location, there are a number of dairy farms around the site and the area receives more than 2 million visitors annually. More information on this site can be found in Berkowitz *et al*.^56^.

#### Southern Great Plains (SGP), USA

The USA Department of Energy, Atmospheric Radiation, Southern Great Plains (SGP) facility is located in north central Oklahoma. The site is located in an agricultural region with mostly wheat, corn, alfalfa, and hay crops. The closest urban centres are Wichita, Kansas, 113 km north, and Oklahoma City, Oklahoma, 136 km south from the site. More details about the instrumentation and operation of the aerosol observing system can be found in^22^ and previous aerosol hygroscopicity results have been presented in Jefferson *et al*.^57^ and Sheridan *et al*.^22^.

#### Trinidad Head (THD), USA

Trinidad Head, California, is located 320 km north of the San Francisco Bay area and 320 km miles south of Eugene, OR and 0.5 km from the Pacific Ocean. This site is located relatively far from large local or regional sources of anthropogenic pollution. THD was established in 2002 at the start of a 1-month intensive field campaign (Intercontinental Transport and Chemical Transformation, ITCT 2K2^58^). The objective of establishing this site was to study aerosol properties entering the USA before they were influenced by North America pollution sources. The site continued as a NOAA monitoring site after the ITCT 2K2 project, but over time instruments were progressively removed, and the site was closed in June 2017.

#### Granada (UGR), Spain

Granada is a medium-sized city in south-eastern Spain. It is situated in a valley surrounded by mountains. The sampling site is located at the Andalusian Institute for Earth System Research (IISTA-CEAMA, University of Granada) in the southern part of the city and it is less than 500 m away from a highway that surrounds the city. The main local aerosol source is road traffic, with influences from domestic heating and biomass burning sources during winter. Results of the relative humidity dependence of aerosol light-scattering at UGR have been reported by Titos *et al*.^25^.

#### Yosemite (YOS), USA

Measurements were conducted at the Interagency Monitoring of Protected Visual Environments (IMPROVE) monitoring site on Turtleback Dome in Yosemite National Park. This site is located on the south rim near the west entrance of Yosemite Valley, in central California. During the study, the site was highly impacted by both fresh and aged carbonaceous aerosols^59^ originating from wildfires burning locally and in Oregon as well as emissions from California’s central valley and the urban areas of San Francisco and San José, located around 250 km to the west. Previous results on aerosol hygroscopicity at the site can be found in Malm *et al*.^60^.

#### Ny-Ålesund (ZEP), Norway

The Zeppelin observatory is located at 475 m a.s.l. on Zeppelin mountain close to the settlement of Ny-Ålesund on the island of Spitsbergen. It is a pristine site characterized by low levels of particle concentrations and typical Arctic aerosol. The clean conditions are dominant in an area where no local sources or long-range transport of aerosols are observed during the period of the year when the tandem nephelometer was deployed (July–October 2008). More details regarding instrumentation and previous results can be found in Zieger *et al*.^61^.

### Overview of different instrumentation designs

All but one system considered in this study consisted of two integrating nephelometers, one operating under low-RH conditions (DryNeph) and the other operated downstream of a humidifier and thus measuring at programmable RH (WetNeph). The exception is Finokalia’s system, in which the WetNeph measured at pseudo-ambient conditions rather than using a humidifier to control humidity conditions.

**Fig. 2:**
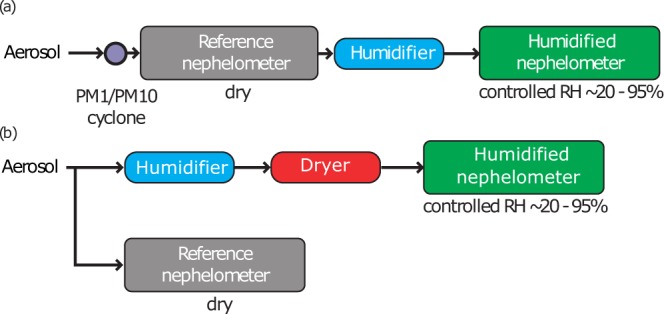
Overview of the two main instrumental designs. Aerosol particles enter through the common station inlet (with station-dependent size cuts and at dry conditions) into the instrumental set-up. (**a**) NOAA design and (**b**) PSI design.

Figure 2 shows a schematic view of the two most common tandem nephelometer designs (the ‘NOAA design’ which was deployed at 17 sites and the ‘PSI design’ which was deployed at 6 sites). Both instruments designs were compared at FKB and further information can be found in Fierz-Schmidhauser *et al*.^23^. For all sites, the reference nephelometer (DryNeph) is run at low RH conditions to measure the particle light scattering coefficient at dry conditions as a reference, while the second nephelometer (WetNeph) measures *σ*_sp_ at varying and elevated RH conditions (RH cycles or scans). The ‘NOAA design’ and the ‘PSI design’ are briefly described below. Additionally, three sites (UGR, FIK and YOS) developed their own tandem nephelometer designs and we provide relevant details of those as well. Table 1 indicates which type of system was deployed at each site and Table 2 shows information about the instrument design.

**Table 2:** Instrument design and data handling.

Station ID	Neph type	Initial drying	Dilution	RH wet	Sensor location	Humidifier system	RH Scan time (h)	Cut size	PM switch (h)
APP	T	Heater	No	Dew point	Downstream	Humi. only	0.5	PM_10_, PM_1_	0.25
BRW	T	Heater	No	Dew point	Downstream	Humi. only	0.5	PM_10_, PM_1_	0.5
CBG	T	Heater	No	Dew point	Upstream	Humi. only	0.5	PM_5_, PM_1_	0.5
CES	T	Drier	No	In neph	—	Humi. + dryer	1.5	PM_10_	—
FIK	E + R	Drier	No	Before neph	—	No	12	PM_1_	—
FKB	T	Heater	No	Dew point	Upstream/Downstream	Humi. only	0.5	PM_10_, PM_1_	0.5
GRW	T	Heater	No	Dew point	Downstream	Humi. only	0.5	PM_10_, PM_1_	0.5
GSN	T	Heater	No	Dew point	Upstream	Humi. only	0.5	PM_10_, PM_1_	0.1
HFE	T	Heater	Yes	Dew point	Downstream	Humi. only	0.5	PM_10_, PM_1_	0.5
HLM	T	Heater	No	In neph	—	Humi. only	0.5	PM_10_, PM_1_	0.5
HYY	E	No	No	In neph	—	Humi. + dryer	1.5	None	—
JFJ	T	No	No	In neph	—	Humi. + dryer	1.5	None	—
KCO	T	Drier + heater	No	Dew point	Upstream	Humi. only	0.5	PM_10_, PM_1_	0.1
LAN	T	Drier	Yes	Dew point	Downstream	Humi. only	0.5	PM_10_	—
MAO	T	Drier	Yes	Dew Point	Downstream	Humi. only	0.5	PM_10_, PM_1_	0.5
MEL	T	Drier	No	In neph	—	Humi. + dryer	1.5	PM_10_	—
MHD	T	No	No	In neph	—	Humi. + dryer	1.5	None	—
NIM	T	Heater	Yes	Dew point	Upstream	Humi. only	0.5	PM_10_, PM_1_	0.5
PGH	T	Heater	No	Dew point	Downstream	Humi. only	0.5	PM_10_, PM_1_	0.5
PVC	T	Heater	No	Dew point	Downstream	Humi. only	0.5	PM_10_, PM_1_	0.5
PYE	T	Heater	No	In neph	—	Humi. only	0.5	PM_10_, PM_1_	0.5
SGP	T	Heater	No	Dew point	Upstream/Downstream	Humi. only	0.5	PM_10_, PM_1_	0.1 or 0.5
THD	T	Heater	No	In neph	—	Humi. only	0.5	PM_10_, PM_1_	0.1 or 0.5
UGR	T	No	No	In neph	—	Humi. only	0.5	None	—
YOS	R	Drier	No	Before neph	—	Humi. + dryer	12	PM_2.5_	—
ZEP	T	No	No	In neph	—	Humi. + dryer	1.5	None	—

In nephelometer type column: T = TSI Inc., Model 3563 (*λ* = 450, 550, 700 nm); E = Ecotech Aurora 3000 (*λ* = 450, 525, 635 nm) and R = Radiance Research model M903 (*λ* = 532 nm at FIK and *λ* = 530 nm at YOS). Sensor location with respect to WetNeph.

#### NOAA design

The tandem nephelometer was deployed at several NOAA-FAN sites (except UGR), DOE/ARM sites (SGP plus the various ARM Mobile Facility deployments) and also at LAN. These systems consist of the two nephelometers (DryNeph and WetNeph) connected in series with the humidifier between them (see Fig. 2a). Prior to passing through an aerosol impactor size cut, the sample air is dried via gentle heating as needed in order to maintain RH < 40%. The heating will depend on the site and the season and typically, temperature changes from sample heating varies from 0 to 10 °C. At some sites with high aerosol loading, additional drying of the sample air was accomplished by diluting the sample with filtered dry air (see Table 2). In order to minimize transmission loss of coarse-mode particles, the system air flow was controlled to 30 lpm, making RH control of the high flow sample air a challenge.

After the impactors, the sample air flows through a reference nephelometer (DryNeph) where the *σ*_sp_(RH_dry_) is measured. The sample air exiting the DryNeph then enters the humidifier which is used to expose the particles to a controlled and elevated RH environment typically between 40 and 85% RH. The humidified air stream then enters the second nephelometer (WetNeph) where *σ*_sp_(RH) is measured as a function of RH. One RH cycle (increasing and decreasing RH) is performed on an hourly basis with the inlet size cut alternating between 10 and 1 *μ*m (aerodynamic diameter) over the course of the hourly cycle at different time intervals depending on the site (see Table 2).

The NOAA system exclusively used one nephelometer type (TSI Inc., Model 3563) for both DryNeph and WetNeph. This instrument measures light scattering and backscattering at *λ* = 450, 550 and 700 nm. The set-up has changed slightly over the years since the initial deployment of a NOAA design tandem nephelometer system at SGP in 1998^22^. One important change over the 20 years of NOAA tandem nephelometer operation was the placement of the RH sensor used to control the humidifier. Originally, this RH sensor was placed at the humidifier outlet. Because of the sharp temperature gradient at the humidifier outlet, this sensor was eventually moved to a more stable RH region at the WetNeph exit.

The NOAA design strategy balances ease of operating with minimal perturbation of the ambient aerosol characteristics. All of the NOAA sites were operated remotely 24/7 with minimal technical service. The humidifier operated in a hydration mode scanning from low to high RH. In order to avoid volatilization of weak acids, the sample heating was regulated to maintain a maximum RH of 40%. One disadvantage of the high flow (30 lpm) NOAA system is that the RH range of the humidifier scan is limited by the ability of the humidifying system to overcome the ambient dew point. At low dew point conditions the humidifier is unable to reach a high RH range. At high dew point conditions the humidifier RH does not extend low enough to capture a minimum in the DryNeph/WetNeph ratio. As described below, the software fits of the data compensated for some of these limitations by specifying boundary conditions to the RH range. More detailed descriptions of this design can be found in Carrico *et al*.^62^, Titos *et al*.^29^, Jefferson *et al*.^57^, and references therein.

#### PSI design

The PSI design has been deployed on a campaign basis at six different sites in Europe within the European Community (EC) projects EUSAAR and GEOmon. In this design, the two nephelometers are operated in parallel. The WetNeph, which is preceded by a humidifying and drying system (see Fig. 2b), measures the *σ*_sp_ at humid conditions. This design allows measurement of both the lower (deliquescent) and upper (efflorescent) branches of the hysteresis curve. A complete humidogram or RH cycle usually took 3 hours: during the first 1.5 hours the aerosol is humidified and RH increases (hydration) and during the last 1.5 hour the aerosol is humidified followed by an active drying (dehydration).

The PSI design utilises multiple calibrated RH sensors located at different points within the system, including inside the nephelometer. Additionally, a dew point sensor measures the dew point temperature. In this design, aerosols encounter the highest RH after passing through the humidifier. The RH is then lowered in the dryer and further lowered inside the nephelometer (due to a ~1 °C temperature increase caused by heating from the nephelometer lamp). A detailed description of this design can be found in Fierz-Schmidhauser *et al*.^23^ and Zieger *et al*.^16^.

Depending on the field site and general inlet conditions, the PSI system measured particles with an aerodynamic diameter lower than 10 *μ*m (PM_10_) or the whole sample air (no size cut), see Table 2. TSI Model 3563 nephelometers were used at all sample sites except HYY, where both nephelometers were replaced by newer LED-based instruments (Ecotech Pty Ltd., Aurora 3000). The Ecotech nephelometers measure at slightly different wavelengths (450, 525, 635 nm) and are less influenced by heat effects from the nephelometer lamp^26^. The internal Kalman filter setting of the instrument was only used during calibration of the nephelometer.

The PSI design includes some major improvements relative to some earlier designs. Firstly, the RH inside the WetNeph is measured by one of the calibrated RH sensors installed directly into the sample volume (as opposed to the manufacturer’s internal *T*/RH sensor relied on in many other humidograph systems). Additionally, an air-cooled infra-red filter is placed in front of the nephelometer halogen light source to minimize changes in sample volume RH due to heating from the lamp. Another feature is that the PSI design can also be operated to measure the upper branch of the hysteresis curve^12^.

#### Other designs: UGR, FIK and YOS

The design at UGR consists of two nephelometers (TSI Inc., Model 3563) sampling from the same inlet, and measuring in parallel. There is no heater/dryer upstream of the instruments to ensure low RH in DryNeph. However, due to the arid conditions in Granada the RH in DryNeph was typically <40%, with a mean value of 20%. The sample air for WetNeph flows through a humidifier, which performs increasing/decreasing RH scans on a 30-min basis, before entering the instrument. There are four *T*/RH sensors, three associated with the WetNeph (located before the humidifier, after the humidifier and inside the nephelometer) and another sensor placed inside the DryNeph (although this last sensor was not operative over the entire measurement period). Further technical details on this system can be found in Titos *et al*.^25^.

For the FIK site, the University of Crete has performed several campaigns measuring particle light scattering as a function of RH since 2009. Two nephelometers are connected in series with a drier between them. The first nephelometer (Radiance Research Model 903, wavelength = 532 nm) serves as WetNeph and measures scattering at pseudo-ambient conditions, performing one cycle per day. The sample air then passes through a diffusion dryer to the second nephelometer (Ecotech Aurora 1000, wavelength = 525 nm) acting as DryNeph. The nephelometers were operated with the Kalman filter off since Finokalia is a remote marine site and rapid variations in the scattering coefficient are not expected to occur except during long range transport events. This study focused on the measurements in 2012 when both nephelometers measured with a PM_1_ size cut.

The USA National Park Service utilized a tandem nephelometer system operating two Radiance Research M903 nephelometers (530 nm) as DryNeph and WetNeph in parallel. The DryNeph was dried to low RH using a drying system and the humidity inside the WetNeph was controlled by two sample air conditioners (which could act as humidifiers or driers) operated in series. In this system it takes between 2 and 3 hours to complete a full RH cycle (only one RH cycle is done each day). When the first conditioner was used as a drier and the second conditioner was used as a humidifier, the deliquescent *f*(RH) can be measured. Both nephelometers were fitted with 2.5 *μ*m cyclone inlets. An in-depth description of this design can be found in Malm *et al*.^60^.

### Calibration of instruments

#### Nephelometer calibrations

There are three standard operating procedures used to ensure the quality of the nephelometer measurements^63^: (1) filtered air checks to obtain the background scattering which is then subtracted from the measured scattering automatically by the instrument; (2) calibration checks to measure instrument response on filtered air and CO_2_ (or another gas with known scattering characteristics) in order to check that the current calibration is still valid; and (3) full instrument calibration which is similar to a calibration check but results in a change of the calibration coefficients in the nephelometer firmware.

NOAA systems performed 5-min filtered air checks on an hourly basis and calibration checks (filtered air and CO_2_) on a weekly to monthly basis. Full calibrations with filtered air and CO_2_ and instrument maintenance (cleaning, inspection, PMT voltage adjustment etc.) were only performed when an instrument scientist was present (i.e., typically on a semi-annual to annual basis).

The nephelometers in the PSI system were calibrated with filtered air and CO_2_ at the beginning of each field campaign, while calibration checks were performed on an irregular basis during the campaign and at the end of the campaign. Filtered air checks were done at least on a daily basis and the nephelometers were also intercompared at dry conditions.

For the UGR system, filtered air checks in both nephelometers were performed hourly. Full calibration with filtered air and CO_2_ and maintenance of the nephelometers (including cleaning and inspection) was performed approximately 4 times per year. Intercomparison of the two nephelometers was performed periodically to check the consistency between the instruments.

The University of Crete in Finokalia performed calibrations (with CO_2_ as a span gas) and checks every 6 months and filtered air checks on a weekly basis. During the 6-month checks, both nephelometers were intercompared while measuring in parallel at the same conditions.

At YOS, filtered air and Freon 134a (a common refrigerant gas with known scattering characteristics^63^) were used to perform full calibrations on an almost daily basis. These frequent calibrations made it unnecessary to carry out filtered air and calibration checks of the nephelometers as calibrations would not be expected to shift over the course of a day.

#### Hygroscopicity-related calibrations

The operation of a humidograph system requires attention to technical detail and calibration^16,23,27^. Specifically, the system RH sensors need to be calibrated frequently to assure that the RH in the system is well characterized. Additionally, optical closure calculations using lab-generated aerosols of known size and composition should be carried out to assess the performance of the system^16,23^. Finally, particle losses and instrumental differences at low RH conditions between the dry and wet nephelometers should be characterized for all the sites.

In the NOAA system, *T*/RH sensors were calibrated on a semi-annual to annual basis. Particle losses in the system were assessed by running the humidifier at low RH conditions and comparing scattering coefficients measured by the two nephelometers. No optical closure calculations were performed on the humidograph system (WetNeph) measurements, although successful optical closure (based on measured size distributions and assumed chemistry) has been performed for DryNeph measurements in several of these systems.

The *T*/RH sensor of the PSI system were calibrated with unsaturated salt solutions, while the light scattering coefficients at prescribed RH in the PSI system were validated using monodisperse or polydisperse salt measurements of ammonium sulphate and sodium chloride generated in the laboratory or in the field (see Fierz-Schmidhauser *et al*.^23^ for more details). The RH is calibrated by comparing its deliquescent and/or efflorescence values expected for salts of known composition. The light scattering coefficients at multiple RH values are compared with the theoretical light scattering coefficients calculated using Mie Theory and the measured particle number size distribution^16,23,26^. Additionally, particle losses in the humidifier were characterized at low RH conditions.

In the UGR system, *T*/RH sensors were calibrated frequently using unsaturated saline solutions of known RH at three calibration points (20, 60, 80%). Additionally, the *T*/RH sensors were periodically intercompared. The system RH was checked by generating and sampling salts of known deliquescence RH^25^. However, a closure study based on the salt measurements was not performed. Particle losses in the humidifier were characterized at low RH conditions and are dependent on the aerosol type, with the highest differences observed under desert dust intrusions, denoting higher losses for larger particles.

At FIK the *T*/RH sensors were not calibrated and losses in the drier were not determined. Closure studies were carried out in Kalivitis *et al*.^35^ where both dry and ambient aerosol light scattering coefficients were reconstructed based on chemical composition (using as main components ammonium sulphate and organic matter for no dust event days) and mass scattering efficiencies. Measured and reconstructed daily averages showed good agreement (*R*^2^ ≥ 0.8).

In Yosemite, calibration and comparisons between the *T*/RH sensors was performed before and at the end of the field campaign. The dry and wet nephelometers were operated under dry conditions for extended time periods daily to assess differences in scattering coefficient between the two instruments. Comparisons were also made between the humidograph system nephelometers and ambient Optec nephelometers to assess particle losses through the inlets. Additional optical closure calibration was done using ammonium sulphate.

### Data handling and harmonization of data sets

**Fig. 3:**
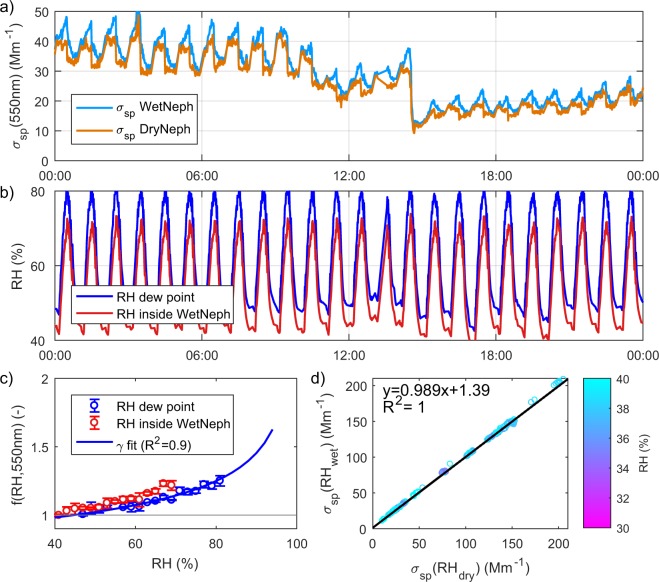
Example of measured data at Manacapuru (MAO) on 18th of October, 2014. (**a**) PM10 Aerosol light scattering coefficients measured by dry and wet nephelometers. (**b**) RH measured by sensor in volume of the wet nephelometer (red) and RH calculated from dew point using an external *T*/RH sensor and internal *T* sensor (blue). (**c**) Measured humidogram of *f*(RH, *λ* = 550 nm) at 11 am, where the error bars denote the standard deviation and the solid blue line is the gamma fit to the data when the dew-point-based RH is used (data *Level 1*). (**d**) Scatter plot of *σ*_sp_(RH_wet_) vs. *σ*_sp_(RH_dry_) for RH <40%, black line denotes linear regression fit.

Below, we describe the general data handling procedure carried out to develop a harmonized data set of RH-dependent *σ*_sp_ and *f*(RH). In Fig. 3, we show an example of the time series of the scattering coefficients and RH measurements at Manacapuru on the 18^th^ of October, 2014, as well as an example of one of the corresponding humidograms for this day (between 11 to 12 am) and the correlation of *σ*_sp_(RH_dry_) and *σ*_sp_(RH_wet_) for different RH values, to guide the reader through the data processing. The processing flow and the products corresponding to each data level are shown in Table 3. Many of the nephelometers also measure aerosol backscattering coefficient (*σ*_bsp_). The backscattering coefficient provides an indication of the angular dependence of light scattering and can be used to derive parameters such as up-scatter fraction and asymmetry parameter^64^. Where this measurement was available in both the DryNeph and WetNeph, the same data handling procedure was followed to process the backscattering data and develop a harmonized data set of RH-dependent *σ*_bsp_. For clarity, we only use the term ‘scattering’ in what follows to encompass both total and backscattering.

**Table 3:** Overview of data levels, applied corrections and corresponding products.

Level	Applied corrections	Products
Raw Data	none	*σ*_sp_(RH), *σ*_bsp_(RH), *σ*_sp_(RH_dry_), *σ*_bsp_(RH_dry_), *T*/RH
Level 0	1-min values. RH sensor correction. Dilution corrections	*σ*_sp_(RH), *σ*_bsp_(RH), *σ*_sp_(RH_dry_), *σ*_bsp_(RH_dry_), *T*/RH
Level 1	1-min values. Data screening: site managers input and time series evaluation. Corrections: angular non-idealities, STP, losses in humidifier, 10-min moving average of *σ*_sp_(RH_dry_) time series	*σ*_sp_(RH), *σ*_bsp_(RH), *σ*_sp_(RH_dry_), *σ*_bsp_(RH_dry_), *f*(RH), *f*_*b*_(RH), *T*/RH, QF
Level 2	1-, 3-, 6- or 12-h values. Interpolation to RH_wet_ values between 30% and 95% at 5% intervals. Fitting of 1-min data. Data screening: RH_dry_ < 40%, RH scan interval >30% and goodness of fit >0.5 or 0.8	*σ*_sp_(RH), *σ*_bsp_(RH), *f*(RH = 85%/RH_dry_), *f*_*b*_(RH = 85%/RH_dry_), *f*(RH = 85%/RH_dry_ = 40%), *f*_*b*_(RH = 85%/RH_dry_ = 40%), Uncertainties, QF

*σ*_sp_(RH): wet particle light scattering coefficient, *σ*_bsp_(RH): wet particle light backscattering coefficient, *σ*_sp_(RH_dry_): dry particle light scattering coefficient, *σ*_bsp_(RH_dry_): dry particle light backscattering coefficient, *f*(RH): light scattering enhancement factor, *f*_*b*_(RH): light backscattering enhancement factor, QF: quality flag.

Data from all sites have been gathered and re-analysed in a standardised way. This harmonization is crucial to intercompare the different sites with their individual instrument characteristics.

The processing starts with the raw data provided by each site mentor/site manager to which we then apply standard corrections using identical methodology. The measurements during filtered air checks and calibration of the instruments are not included in our dataset. The raw data consists of the high frequency measurements with 1 minute timebase. A first homogenization step is necessary in the case of ZEP and HLM, since these data are recorded at higher frequency (1 sec and 20 sec timebase, respectively) and needed to be averaged to the 1 minute timebase. Additionally, while the dilution correction necessary for HFE, MAO and NIM is already incorporated into the raw data provided by the site operator, a dilution correction is applied to the LAN data set. The RH calibrations for the PSI systems are applied during this initial processing phase, too (the RH calibrations for other systems are already incorporated in the raw data). This preliminary, homogenized dataset corresponds to *Level 0* data.

When the *Level 0* dataset is finalised, in-depth data screening is carried out in order to obtain the *Level 1* data. As a first step, this consists of removing data during invalid periods and during system malfunctions (i.e., as indicated in each site logbook or the editing directives from the data provider(s)). The time series of the dry and wet scattering coefficients as well as RH and *T* values for each site are then further inspected in order to identify possible outliers and additional questionable data periods that had not been flagged during the data provider’s quality control processing. Valid measurements are flagged with the quality flag (QF) set to 0 and, for invalid measurements, the quality flag is set to 2. Periods at PSI sites when the humidograph is not scanning RH values, but rather operating at a constant high or low RH are included with QF = 0 in *Level 1* data if no other problem is detected in the quality control.

After identifying the good (QF = 0) data, several corrections are applied. First, the nephelometers are corrected for angular truncation and illumination non-idealities. For the TSI and Radiance Research nephelometers, the correction scheme proposed by Anderson *et al*.^63^ is used, while the correction scheme developed by Müeller *et al*.^65^ is applied to the Ecotech Aurora nephelometers. Next, an adjustment to standard temperature and pressure (STP, *T* = 273.15 K and *P* = 1013.25 hPa) is applied to all values of *σ*_sp_. Figure 3a shows the *Level 1* time series of *σ*_sp_ as an example.

In order to account for potential particle losses within the instrument system and to identify discrepancies between the two nephelometer calibrations, the linear regression of *σ*_sp_ in the DryNeph and *σ*_sp_ in the WetNeph when both instruments are operating at similar low RH values (typically 20 < RH < 50%) is calculated. As an example, Fig. 3d shows *σ*_sp_(RH_wet_) versus *σ*_sp_(RH_dry_) measured in MAO colour-coded for RH to illustrate this point. The derived correction is then applied to the WetNeph *σ*_sp_ and ranges between 5 and 15% for most of the sites, with the highest value in PYE of 18% (for total scattering and 550 nm). UGR is frequently affected by Saharan dust outbreaks, where we have observed higher losses in the humidifier for larger particles and therefore we have applied different correction factors for dust-free and dust conditions at this site. Finally, a 10 minute moving average (11 measurements) is applied to *σ*_sp_(RH_dry_) in order to reduce the influence of noise and outliers. This averaging helps to minimize noise in the *f*(RH) calculations especially during periods of extremely low *σ*_sp_(RH_dry_). This moving average is especially necessary for measurements at pristine sites with very low particle concentrations (e.g., ZEP, JFJ, BRW), but is applied to all data sets for consistency. The corrected *Level 1* data is used for the calculations of the scattering enhancement factor (*f*(RH)) also provided in the *Level 1* datafile.

The *Level 2* data includes the particle light scattering coefficients for RH values ranging from 30% to 95% at intervals of 5%. Each of these *σ*_sp_(RH) values are obtained by interpolating between *Level 1* scattering measurements obtained at the closest two RH values bracketing the desired 5% RH interval. The results in *Level 2* are given in an averaged (1, 3, 6 or 12 hours) data file with up to 20 interpolated scattering values (one for each RH interval) representing the RH scan for each humidogram. Data points are set to the missing value code when measurements are not available for interpolation.

### Determining the sample volume RH

The RH inside the dry and wet nephelometers is a critical parameter for the precise determination of *f*(RH). Here we call these values RH_dry_ and RH_wet_. For all sites the RH_dry_ is always the RH measured by the manufacturer’s sensor inside the DryNeph (or, in the case of Radiance Research nephelometers, at the exhaust of the DryNeph). RH_wet_ is determined in different ways depending on the system design as described below. Table 2 lists the method used to calculate RH_wet_ for each site.

For the PSI systems we utilise the additional installed and calibrated RH sensor inside the nephelometer sample volume as RH_wet_. Zieger *et al*.^16^ emphasised the need for salt calibrations to determine the exact RH at the point of light scattering detection inside the WetNeph. In addition, one should keep in mind that the exact deliquescence RH measured by the WetNeph may not be the same as the thermodynamic deliquescence RH due to temperature differences between humidifier and subsequent nephelometer where the light scattering is being measured [see^23^]. Like the PSI systems, the UGR system also relies on a calibrated RH sensor inserted in the WetNeph sample volume, rather than using the manufacturer’s internal *T*/RH sensor. RH_wet_ values for YOS and FIK are obtained from an external RH sensor downstream of the WetNeph. These two sites utilised Radiance Research nephelometers which are less subject to the lamp heating issues that occur with TSI nephelometers.

For the NOAA design systems, the determination of RH_wet_ is less straightforward as two potential RH_wet_ values are evaluated. The first approach is to use the RH measured by the manufacturer’s internal *T*/RH sensor in the WetNeph sample volume. The second approach is to calculate the sample dew point temperature using a calibrated external *T*/RH sensor (placed upstream or downstream of the WetNeph depending on the site, see Table 2) and then use that dew point value to calculate the RH inside the nephelometer sample volume based on temperature measured by the manufacturer’s internal *T* sensor. As Fig. 3b shows, discrepancies between the RH values calculated by these two methods may exist. One possible reason is drift in the manufacturer’s calibration of the internal RH sensor^25^. Another reason is that the internal *T*/RH sensor is located in an instrument wall cavity outside of the central sample airflow. As such, the sensor is susceptible to the thermal inertia of the instrument wall as well as radial RH differences between the centre flow and wall. To assure the best choice for RH_wet_, time series of RH measured by the manufacturer’s internal *T*/RH sensor and RH calculated with the dew point are analysed. If no problem appears throughout the entire measurement period and the RH values agree, the RH calculated based on dew point is selected as RH_wet_. For HLM, the *T*/RH sensor placed upstream of the WetNeph was affected by the humidifier and the RH values exhibit large variability. For THD, there is a period where the external *T*/RH sensor did not measure correctly. In these two cases, RH measured inside the WetNeph is selected as the best choice for the RH_wet_.

### Determination of *f*(RH)

Using the corrected *Level 1* data the the total and back scattering enhancement factors, *f*(RH) and *f*_b_(RH), can be now obtained using Eq. 1. Each humidogram, i.e., the *f*(RH) values as a function of RH_wet_ for each individual scan in the *Level 1* data, can be numerically parametrised using a variety of equations (for a summary see Titos *et al*.^27^). The *Level 2* data presented here (see example in Fig. 3c), uses a variation of the most common fit equation initially introduced by Kasten *et al*.^66^. This is a two parameter fit equation where parameter *a* represents the intercept at RH = 0% and parameter *γ* is an indicator of aerosol hygroscopicity:

2 $$f(RH)=a{(1-RH/100{\rm{ \% }})}^{-\gamma }.$$

Several sites are not able to maintain suitably dry conditions inside the DryNeph (i.e., the RH of the dry nephelometer is occasionally (or even frequently), higher than 40%). Calculated *f*(RH) values for time periods when RH_dry_ > 40% are flagged as invalid (QF = 2) in the *Level 1* data.

Constraints are imposed on each humidogram in order to obtain valid fits. First, only those humidograms spanning an RH_wet_ range larger than 30% in the WetNeph are included in the *Level 2* data. Since most humidograms start at RH larger than 30–40%, this means that fits typically cover RH ranges at least up to 70% (and usually higher). Humidograms spanning a narrower RH interval are flagged as invalid in the *Level 2* file. Additionally, a goodness-of-fit criterion is applied such that humidogram fits with a *R*-squared value less than 0.5 are also flagged as invalid in the *Level 2* file. A stricter goodness of fit requirement is used for Hyytiälä, Jungfraujoch (*R*-squared value threshold was set to 0.7 and 0.8, respectively) where higher variability is observed in the RH scans, mostly during summer months due to the uplift of air masses and the atmospheric boundary layer. At FIK, which used pseudo-ambient conditions rather than controlling RH, and at YOS, which scanned over long periods, 12 hours of measurements are taken into account for each humidogram to increase the number of scans meeting the RH_wet_ range larger than 30% criterion. To avoid possible errors produced by sharp changes of air masses over the 12h period, the *R*-squared value threshold for FIK and YOS is set to 0.9 to select humidograms representing relatively constant air masses.

In the *Level 2* data file, *f*(RH = 85%/RH_dry_) and *f*(RH = 85%/RH = 40%) are provided together with its relative uncertainty. The value of *f*(RH_wet_/RH_dry_) has been calculated in two ways. First, and as main product, it is calculated with the measured reference scattering (DryNeph) represented by *σ*_sp_(RH_dry_) where RH_dry_ is the measured RH in the DryNeph (within the range of 0–40%). Secondly, for comparison reasons, *f*(RH_wet_/RH = 40%) is derived with the reference scattering coefficient *σ*_sp_(RH = 40%) obtained from the interpolated *Level 1* data at RH = 40% (WetNeph). This was done only for humidograms where the RH scan time was below 1.5 hours, therefore excluding sites like YOS and FIK. It should be noted that the second approach does not account for possible rapid changes in aerosol load. All these quantities are given for the three nephelometer wavelengths and for the total and backscattering coefficients.

### Uncertainty analysis for *σ*_sp_(RH) and *f*(RH)

The uncertainty associated with the light total and back scattering coefficients has been calculated following the methodology developed in Sherman *et al*.^31^, explained in detail in their supplementary materials. Briefly, major sources of uncertainty in *σ*_sp_ and *σ*_bsp_ measured by the nephelometer are: instrumental noise, uncertainty in the nephelometer calibration, nephelometer calibration variability, uncertainty in the correction for nephelometer angular non-idealities, and uncertainty in correcting light scattering to standard temperature and pressure (STP) conditions. In this study, in order to represent the range of aerosol conditions at the 26 sampling sites, calculations have been performed for different levels of aerosol loading, with *σ*_sp_ values of 5, 50 and 200 Mm^−1^ (for *σ*_bsp_ the loading values used were a factor of 10 lower), 1-minute averaging time, pressure values ranging from 700 to 1013 hPa, temperature values ranging from 293.15 to 303.15 K and differentiating between no size cut and PM_1_ particles since the truncation correction uncertainty is different for these two subsets of particles^63^.

The uncertainty associated with *σ*_sp_(RH_wet_) and *σ*_bsp_(RH_wet_) can then be calculated by error propagation using Eq. 2, where the absolute uncertainty associated with the measurement of RH is assumed to be 3%, selected as an upper conservative threshold at high RH for the RH sensors commonly used in the different designs of the humidified tandem nephelometers. Uncertainties in *σ*_sp_(RH_wet_), *σ*_bsp_(RH_wet_) and *f*(RH) vary depending on aerosol load, RH and hygroscopicity of particles. Calculations are carried out considering RH ranging from 50% to 85% and particles with low and high hygroscopicity, assuming a gamma parameter ranging between 0.2 and 0.9. A summary of uncertainties in *σ*_sp_(RH_dry_), *σ*_sp_(RH_wet_) and *f*(RH) at *λ* = 550 nm is shown in Table 4. Since the observed influence of *T* and *P* on the uncertainty is small, results presented in Table 4 are given for *T* = 20 °C and *P* = 1013 *hPa*.

**Table 4:** Relative uncertainties (%) in total (back) *σ*(RH_dry_), *σ*(RH_wet_), and *f*(RH) (%).

Hygroscopic growth	Aerosol load (Mm^−1^)	RH = 0%	RH = 50%		RH = 85%	
	*σ*_sp_(*σ*_bsp_)	Δ(*σ*(RH_dry_))	Δ(*σ*(RH_wet_))	Δ(*f*(RH))	Δ(*σ*(RH_wet_))	Δ(*f*(RH))
**PM**_**1**_						
*γ* = 0.2	5 (0.5)	10.0 (36.8)	11.2 (38.0)	15.0 (52.9)	14.0 (40.8)	20.5 (66.8)
	50 (5)	7.5 (8.7)	8.7 (9.9)	11.5 (13.2)	11.5 (12.7)	16.3 (18.3)
	200 (20)	7.4 (7.7)	8.6 (8.9)	11.3 (11.7)	11.4 (11.7)	16.1 (16.5)
*γ* = 0.6	5 (0.5)	10.0 (36.8)	13.3 (40.4)	16.9 (54.6)	22.0 (48.8)	27.8 (73.24)
	50 (5)	7.5 (8.7)	11.1 (12.3)	13.4 (15.1)	19.5 (20.7)	23.7 (25.6)
	200 (20)	7.4 (7.7)	11.0 (11.3)	13.3 (13.6)	19.4 (19.7)	23.5 (23.9)
*γ* = 0.9	5 (0.5)	10.0 (36.8)	15.4 (42.2)	18.4 (56.0)	28.0 (54.8)	33.5 (78.3)
	50 (5)	7.5 (8.7)	12.9 (14.1)	14.9 (16.6)	25.5 (26.7)	29.5 (31.4)
	200 (20)	7.4 (7.7)	12.8 (13.1)	14.8 (15.1)	25.4 (25.7)	29.4 (29.8)
**PM**_**10**_						
*γ* = 0.2	5 (0.5)	11.0 (37.0)	12.2 (38.2)	16.4 (53.1)	15.0 (41.0)	22.2 (67.1)
	50 (5)	8.8 (9.4)	10.0 (10.6)	13.3 (14.2)	12.8 (13.4)	18.4 (19.6)
	200 (20)	8.7 (8.5)	9.9 (9.7)	13.2 (12.9)	12.7 (12.5)	18.3 (18.0)
*γ* = 0.6	5 (0.5)	11.0 (37.0)	14.6 (40.6)	18.2 (54.9)	23.0 (49.0)	29.3 (73.6)
	50 (5)	8.8 (9.4)	12.4 (13.0)	15.2 (16.1)	20.8 (21.4)	25.7 (26.8)
	200 (20)	8.7 (8.5)	12.3 (12.1)	15.1 (14.8)	20.8 (20.5)	25.6 (25.3)
*γ* = 0.9	5 (0.5)	11.0 (37.0)	16.4 (42.4)	19.7 (56.2)	29.0 (55.0)	35.0 (78.6)
	50 (5)	8.8 (9.4)	14.2 (14.8)	16.7 (17.6)	26.8 (27.4)	31.5 (32.6)
	200 (20)	8.7 (8.5)	14.1 (13.9)	16.6 (16.3)	26.7 (26.5)	31.4 (321.1)

For PM_10_ and PM_1_ particles at *λ* = 550 nm, for low to high ranges of hygroscopic growth (*γ* = 0.2, 0.6, 0.9), different levels of aerosol load (*σ*_sp_ = 5, 50, 200 Mm^−1^ and *σ*_bsp_ = 0.5, 5, 20 Mm^−1^), T = 20 °C, P = 1013 hPa, and 1 minute averaging time.

Titos *et al*.^27^ calculated the uncertainty of *f*(RH) by Monte Carlo technique, associating an uncertainty of 9.2% with both *σ*_sp_(RH_dry_) and *σ*_sp_(RH_wet_) and considering a range of aerosol loads and hygroscopic growth factors. The results obtained for the uncertainty of low hygroscopic particles, with *γ* = 0.2, increases with RH and varies between 10–15%. For highly hygroscopic particles (for example, for *γ* = 0.9), uncertainty ranged between 15–40% for increasing values of RH. Jefferson *et al*.^57^ also obtained relative uncertainties for *σ*_sp_(RH) using error propagation and a Monte Carlo technique, finding uncertainties associated with wet scattering coefficient between 19.2 and 25.3% for *σ*_sp_(RH_dry_) = 10 Mm^−1^ (and between 9.6 and 18.7% for σ_sp_(RH_dry_) =100 Mm^−1^) and a reference RH_dry_ = 40% for different values of *γ* and RH_wet_. Our results and those reported by Jefferson *et al*.^57^ follow similar behaviour, showing a decrease in relative uncertainty of *σ*_sp_(RH) for increases in aerosol load, decreases of RH, and decreases in hygroscopic parameter *γ*. Therefore, the relative uncertainty of *σ*_sp_(RH) increases for low aerosol loads, which is important especially for polar, clean marine, or mountain sites with predominantly observed low aerosol concentrations.

There may be additional uncertainty related to the different configurations of the humidified nephelometer systems. This relates mainly to the order of the humidifier and dryer, the method of drying (active drying vs. heating), whether calibrations with mono-disperse salt calibrations were performed and the number of sensors used within the system to monitor RH and temperature. The uncertainty contributions related to these configuration differences are difficult to quantify (see Fierz-Schmidhauser *et al*.^23^, for more details). In addition, there might be further and undocumented circumstances possibly affecting measurement reliability and uncertainty resulting from the field operation (e.g., changes in building heating/cooling, system leaks, etc.) as can happen at any long-term monitoring site.

## Data Records

Data records are composed of 339 ASCII files (NASA Ames format) organized in three levels containing the products shown in Table 3. One file is provided per site, size cut, year, and data level.

The files are available on the EBAS Data Portal, accessible through the URL: http://ebas.nilu.no. Individual files are accessible via a search function, including visualization tools. Data has also been deposited to ACTRIS Data Centre^67^ under the following 10.21336/gen.4.

Each file has a number of lines with relevant metadata, followed by the corresponding data products. For metadata information please visit: https://ebas-submit.nilu.no/Submit-Data/Data-Reporting/Templates/Category/Aerosol/Integrating-Nephelometer-Data.

## Technical Validation

**Fig. 4:**
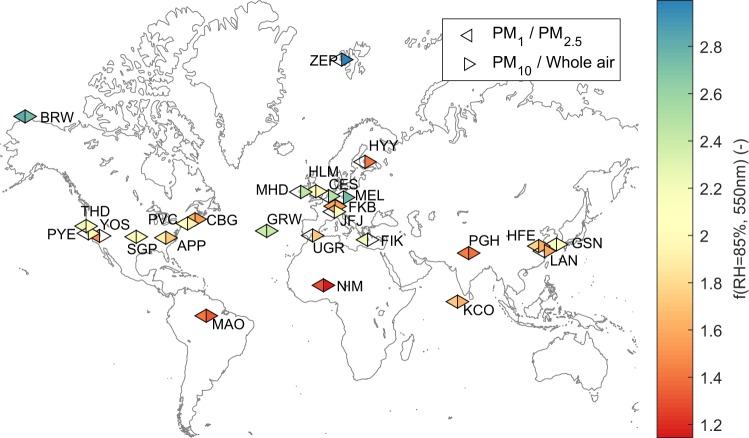
Overview of the sites analysed within this study. Symbols show mean values of *f*(RH = 85%/RH_dry_) for PM_1_/PM_2.5_ (left triangles) and PM_10_/whole-air inlet systems (right triangles).

**Fig. 5:**
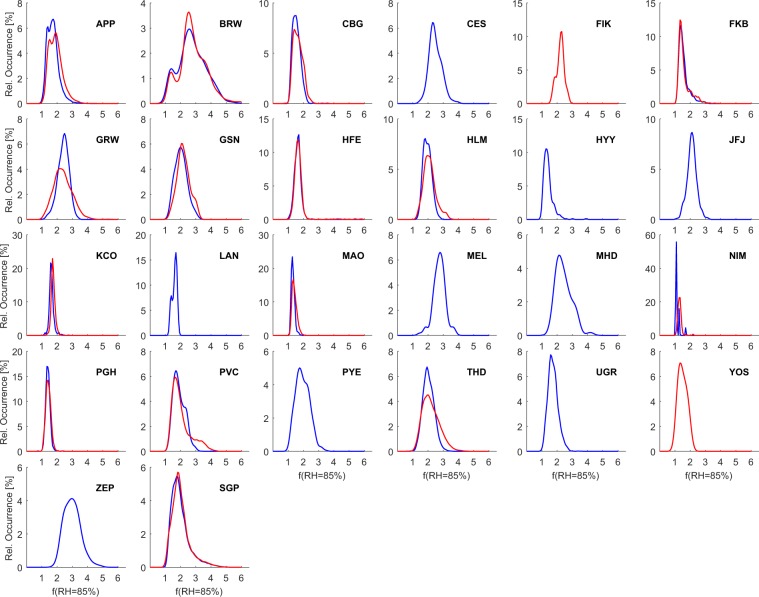
Overview of the analysed data for all 26 sites. Relative frequency of occurrence (%) of *f*(RH = 85%/RH_dry_) calculated from the humidogram data (*Level 2*). Red lines correspond to either PM_1_ or PM_2.5_ and blue lines to either PM_10_ or whole air.

Figure 4 shows the location of the different sites and the mean values of *f*(RH = 85%/RH_dry_) (segregated by size-cut when possible), while Fig. 5 shows the frequency of occurrence of the *f*(RH = 85%/RH_dry_) for different size cuts at the 26 sites. The mean, standard deviation and percentiles (25th, 50th and 75th) are given in Table 5 for total light scattering enhancement factors (*f*(RH = 85%/RH_dry_) and *f*(RH = 85%/RH = 40%)) and in Table 6 for light backscattering enhancement factors (*f*_*b*_(RH = 85%/RH_dry_) and *f*_*b*_(RH = 85%/RH = 40%)). In Table 6 some sites are missing due to the lack of backscattering coefficient measurements (like FIK and YOS) or because their measurements did not meet the quality criteria (like PGH and MAO).

**Table 5:** Summary of average values for total particle light scattering enhancement factor (*Level 2* data).

Station ID	*f*(RH = 85%/RH_dry_)												*f*(RH = 85%/RH = 40%)											
	PM_10_ or whole-air						PM_1_ or PM_2.5_						PM_10_ or whole air						PM_1_ or PM_2.5_					
	P25	P50	P75	Mean	Std	N	P25	P50	P75	Mean	Std	N	P25	P50	P75	Mean	Std	N	P25	P50	P75	Mean	Std	N
APP	1.4	1.7	1.9	1.7	0.4	27627	1.6	1.9	2.2	1.9	0.5	26238	1.5	1.8	2.0	1.8	0.5	16313	1.7	2.0	2.3	2.0	0.5	10644
BRW	2.2	2.7	3.4	2.7	0.9	1956	2.3	2.7	3.4	2.8	0.9	1500	1.9	2.3	2.8	2.4	0.6	1219	1.9	2.3	2.7	2.4	0.6	1080
CBG	1.3	1.5	1.7	1.5	0.3	893	1.4	1.6	1.9	1.6	0.3	913	1.3	1.6	1.8	1.6	0.4	538	1.4	1.6	1.8	1.7	0.4	507
CES	2.2	2.4	2.7	2.5	0.4	657	—	—	—	—	—	—	2.0	2.3	2.7	2.4	0.6	440	—	—	—	—	—	—
FIK	—	—	—	—	—	—	2.1	2.3	2.4	2.2	0.2	46	—	—	—	—	—	—	—	—	—	—	—	—
FKB	1.3	1.5	1.7	1.6	0.3	1875	1.3	1.4	1.7	1.6	0.4	1532	1.3	1.5	1.7	1.6	0.4	1497	1.3	1.5	1.8	1.7	0.5	1077
GRW	2.2	2.4	2.7	2.4	0.4	4383	2.0	2.3	2.8	2.4	0.6	2643	2.1	2.4	2.8	2.5	0.6	1040	2.4	2.8	3.3	2.9	0.6	542
GSN	1.8	2.0	2.3	2.0	0.4	139	2.0	2.2	2.4	2.2	0.4	138	1.8	2.1	2.3	2.1	0.4	58	1.8	2.0	2.2	2.1	0.4	72
HFE	1.5	1.6	1.7	1.6	0.2	2089	1.5	1.6	1.8	1.8	0.8	2075	1.4	1.6	1.8	1.6	0.3	1527	1.5	1.7	1.9	1.7	0.3	1465
HLM	1.8	1.9	2.1	2.0	0.3	499	1.8	2.1	2.3	2.1	0.4	428	1.7	1.9	2.1	2.0	0.4	408	1.7	1.9	2.2	2.0	0.4	202
HYY	1.2	1.4	1.5	1.4	0.3	274	—	—	—	—	—	—	1.4	1.5	1.6	1.5	0.2	72	—	—	—	—	—	—
JFJ	1.9	2.1	2.3	2.1	0.3	216	—	—	—	—	—	—	2.0	2.4	2.8	2.5	0.8	179	—	—	—	—	—	—
KCO	1.55	1.61	1.69	1.63	0.16	615	1.66	1.73	1.81	1.74	0.12	650	—	—	—	—	—	—	—	—	—	—	—	0
LAN	1.48	1.63	1.72	1.60	0.16	605	—	—	—	—	—	—	1.5	1.6	1.7	1.6	0.2	557	—	—	—	—	—	—
MAO	1.23	1.30	1.38	1.31	0.11	797	1.29	1.38	1.50	1.41	0.16	1378	1.17	1.21	1.35	1.23	0.12	6	1.2	1.4	1.7	1.4	0.2	10
MEL	2.5	2.8	3.0	2.7	0.4	99	—	—	—	—	—	—	2.1	2.3	2.7	2.4	0.5	70	—	—	—	—	—	—
MHD	2.0	2.3	2.7	2.4	0.5	61	—	—	—	—	—	—	1.8	2.1	2.8	2.5	0.9	36	—	—	—	—	—	—
NIM	1.07	1.08	1.12	1.14	0.16	19	1.22	1.29	1.35	1.30	0.14	83	1.1	1.2	1.4	1.4	0.5	589	1.2	1.4	1.8	1.6	0.6	467
PGH	1.30	1.38	1.47	1.39	0.15	699	1.31	1.41	1.53	1.42	0.17	788	1.3	1.4	1.6	1.5	0.4	646	1.3	1.4	1.6	1.5	0.3	736
PVC	1.6	1.8	2.2	1.9	0.4	2670	1.6	1.9	2.3	2.0	0.6	2388	1.5	1.7	2.2	1.9	0.5	2275	1.6	1.8	2.3	2.0	0.6	1817
PYE	1.6	1.9	2.3	2.0	0.5	1186	—	—	—	—	—	—	2.0	2.3	2.6	2.4	0.6	336	—	—	—	—	—	—
SGP	1.6	1.9	2.2	2.0	0.6	68290	1.6	1.9	2.3	2.0	0.6	68480	1.6	1.9	2.3	2.0	0.6	53730	1.6	1.9	2.3	2.1	0.6	54613
THD	1.8	2.0	2.3	2.0	0.4	21880	1.8	2.1	2.6	2.2	0.6	21087	1.7	2.0	2.3	2.1	0.7	5870	1.7	2.0	2.4	2.1	0.7	4293
UGR	1.5	1.7	1.9	1.7	0.3	2566	—	—	—	—	—	—	1.6	1.8	2.1	1.9	0.5	1258	—	—	—	—	—	—
YOS	—	—	—	—	—	—	1.3	1.4	1.7	1.5	0.3	25	—	—	—	—	—	—	1.5	1.6	1.7	1.6	0.2	13
ZEP	2.6	3.0	3.3	3.0	0.5	155	—	—	—	—	—	—	2.2	2.5	3.0	2.7	0.8	126	—	—	—	—	—	—

Percentile values (25th, 50th and 75th percentile, called P25, P50 and P75, respectively), mean and standard deviation (Std) of *f*(RH = 85%/RH_dry_) and *f*(RH = 85%/RH = 40%) for the sites analysed in this study. N: Number of valid humidograms. *λ* = 525 nm for FIK and HYY, *λ* = 530 nm at YOS, and *λ* = 550 nm for the rest. Values are separated according to PM_1_/PM_2.5_ and PM_10_/whole-air inlets and are calculated following the specific procedures and data conditions described in the text.

**Table 6:** Summary of average values for particle light backscattering enhancement factor (*Level 2* data).

Station ID	*f*_*b*_(RH = 85%/RH_dry_)												*f*_*b*_(RH = 85%/RH = 40%)											
	PM_10_ or whole-air						PM_1_ or PM_2.5_						PM_10_ or whole air						PM_1_ or PM_2.5_					
	P25	P50	P75	Mean	Std	N	P25	P50	P75	Mean	Std	N	P25	P50	P75	Mean	Std	N	P25	P50	P75	Mean	Std	N
APP	1.2	1.3	1.4	1.3	0.2	13123	1.2	1.3	1.5	1.4	0.2	13607	1.2	1.4	1.5	1.4	0.4	8043	1.3	1.4	1.6	1.5	0.4	5330
BRW	2.0	2.4	2.8	2.4	0.5	295	2.4	2.8	3.1	2.8	0.6	75	1.8	2.1	2.5	2.2	0.5	201	2.0	2.3	2.6	2.3	0.4	66
CBG	1.20	1.29	1.37	1.29	0.13	542	1.20	1.33	1.43	1.32	0.14	548	1.2	1.3	1.4	1.3	0.2	334	1.2	1.3	1.4	1.4	0.2	306
CES	1.5	1.6	1.9	1.7	0.4	501	—	—	—	—	—	—	1.5	1.6	1.9	1.8	0.6	316	—	—	—	—	—	—
FKB	1.4	1.5	1.7	1.6	0.4	205	1.3	1.5	1.6	1.5	0.4	161	1.2	1.4	1.7	1.5	0.5	260	1.3	1.5	1.8	1.6	0.4	171
GRW	1.5	1.6	1.8	1.7	0.2	2475	0.9	1.1	1.4	1.2	0.4	774	1.6	1.9	2.2	2.0	0.6	563	1.8	2.1	2.7	2.3	0.8	234
GSN	1.21	1.26	1.50	1.33	0.17	43	1.22	1.29	1.52	1.34	0.16	31	1.3	1.5	1.5	1.4	0.2	7	1.2	1.3	1.5	1.3	0.2	12
HFE	1.19	1.25	1.32	1.26	0.10	999	1.2	1.2	1.3	1.3	0.7	922	1.2	1.3	1.4	1.4	0.3	638	1.2	1.3	1.5	1.4	0.3	610
HLM	1.3	1.4	1.6	1.5	0.2	284	1.3	1.4	1.6	1.4	0.2	182	1.3	1.5	1.7	1.6	0.4	219	1.3	1.5	1.6	1.5	0.3	73
JFJ	1.56	1.65	1.80	1.68	0.13	9	—	—	—	—	—	—	1.6	1.8	2.0	1.9	0.4	29	—	—	—	—	—	—
KCO	1.26	1.30	1.37	1.31	0.13	492	1.21	1.27	1.37	1.28	0.14	440	—	—	—	—	—	—	—	—	—	—	—	—
LAN	1.18	1.25	1.31	1.24	0.10	597	—	—	—	—	—	—	1.20	1.27	1.36	1.29	0.13	398	—	—	—	—	—	—
MEL	1.71	1.83	1.95	1.83	0.17	77	—	—	—	—	—	—	1.6	1.7	2.0	1.8	0.3	44	—	—	—	—	—	—
MHD	1.6	1.8	2.3	2.0	0.5	29	—	—	—	—	—	—	1.3	1.9	2.2	2.0	0.8	22	—	—	—	—	—	—
NIM	1.1	1.1	1.3	1.2	0.2	4	1.19	1.25	1.28	1.24	0.10	3	1.2	1.3	1.6	1.5	0.6	387	1.3	1.5	2.1	1.8	0.8	255
PVC	1.5	1.6	1.8	1.6	0.2	465	1.6	2.3	2.7	2.2	0.6	87	1.5	1.7	1.9	1.8	0.4	504	1.5	2.0	2.6	2.2	0.8	94
PYE	1.3	1.4	1.6	1.5	0.3	176	—	—	—	—	—	—	1.4	1.6	1.7	1.7	0.4	83	—	—	—	—	—	—
SGP	1.3	1.4	1.6	1.4	0.3	24838	1.3	1.4	1.6	1.5	0.3	24718	1.3	1.4	1.6	1.5	0.4	20156	1.3	1.5	1.7	1.6	0.4	21171
THD	1.4	1.5	1.7	1.5	0.3	14104	1.3	1.6	1.9	1.6	0.5	7002	1.4	1.6	1.8	1.7	0.6	3576	1.3	1.5	1.8	1.7	0.8	1631
UGR	1.2	1.3	1.3	1.3	0.1	547	—	—	—	—	—	—	1.3	1.5	1.7	1.6	0.4	287	—	—	—	—	—	—
ZEP	1.8	2.1	2.6	2.4	1.0	12	—	—	—	—	—	—	1.6	2.3	3.2	2.4	0.9	6	—	—	—	—	—	—

Percentile values (25th, 50th and 75th percentile, called P25, P50 and P75, respectively), mean and standard deviation (Std) of *f*_*b*_(RH = 85%/RH_dry_) and *f*_*b*_(RH = 85%/RH = 40%) for the sites analysed in this study. N: Number of valid humidograms. *λ* = 525 nm for HYY, and *λ* = 550 nm for the rest. Values are separated according to PM_1_/PM_2.5_ and PM_10_/whole-air inlets and are calculated following the specific procedures and data conditions described in the text.

Overall, Arctic and marine sites exhibit the highest values of *f*(RH = 85%/RH_dry_) (median values ranging between 1.5 and 3.0 for PM_10_) and desert, urban and polluted sites the lowest *f*(RH = 85%/RH_dry_) values (ranging from 1.1–1.7 for PM_10_). Mountain and rural sites exhibit a wide range of values (spanning 1.4 to 2.7 for PM_10_). These ranges are consistent with what has previously been reported for *f*(RH = 85%/RH_dry_) as a function of aerosol type, e.g., Titos *et al*.^27^. Slightly higher values are reported for PM_1_ than PM_10_, which is also consistent with previous works (e.g., Carrico *et al*.^6^).

Where possible, results from specific sites in the benchmark dataset have been compared to previous findings reported in the literature for those same sites. Differences between the benchmark dataset values and literature values may occur for several reasons, such as (a) consideration of different measurement periods, (b) applying different and/or additional data screening, (c) analysis procedures and/or (d) segregating by different types of air masses (not done in this benchmark dataset as it requires additional information). Additionally, how *f*(RH) is reported can differ. For example, some authors report *f*(RH = 85%), i.e., the wet scattering at a defined RH (e.g., RH_wet_ = 85%) referenced to the dry scattering at the RH inside the dry nephelometer. Others may report *f*(RH) at defined wet and dry RH values (e.g., RH_wet_ = 85%, RH_dry_ = 40%). Overall, the differences found between our results and those found in literature are within the uncertainty.

The PSI sites have been analysed in Zieger *et al*.^16,26^ and references therein. Values of mean *f*(RH = 85%/RH_dry_) for all atmospheric conditions (i.e., not segregated by air mass type) at CES, JFJ, HYY, MEL, MHD and ZEP were reported as: 2.4 ± 0.4, 2.3 ± 0.3, 1.6 ± 0.2, 2.8 ± 0.4, 2.1 ± 0.3, and 3.2 ± 0.6, respectively. Our values for the same sites and all atmospheric conditions are similar but tend to be slightly lower except at CES, i.e., 2.5 ± 0.4, 2.1 ± 0.3, 1.4 ± 0.3, 2.7 ± 0.4, 2.4 ± 0.5, and 3.0 ± 0.5 for CES, JFJ, HYY, MEL, MHD and ZEP, respectively. Fierz-Schmidhauser *et al*.^46^ gives averages for *f*(RH = 85%/RH_dry_) excluding Saharan Dust Events at JFJ, these values are 2.2 and 1.6 for light scattering and backscattering enhancement factor while in our study we obtain 2.1 ± 0.3 and 1.7 ± 0.1, respectively.

Some other authors have also studied *f*(RH = 85%/RH_dry_). For LAN, Zhang *et al*.^49^ reported a mean value for PM_10_ of 1.6 ± 0.1 which is the same as the value reported in this study. For PGH, Dumka *et al*.^54^ obtained a mean value of 1.3 ± 0.1 for both PM_10_ and PM_1_, similar to our value 1.4 ± 0.2. For the urban site UGR, a mean value (under urban atmospheric conditions) of 1.6 ± 0.3 has been reported by Titos *et al*.^25^, while in our study (for all atmospheric conditions) we find a median value of 1.7 ± 0.3. The rural polluted site of YOS was found to have mean values for PM_2.5_ of 1.3 ± 0.2^60^, while in this study a mean value of 1.5 ± 0.3 is obtained.

Several studies give values for *f*(RH = 85%/RH = 40%). Doherty *et al*.^38^ studied data from the polluted marine site of GSN and found mean values of 2.3 ± 0.6 for PM_10_ and 2.4 ± 0.5 for PM_1_, which are similar to the values obtained in our study of 2.1 ± 0.4 for PM_10_ and PM_1_. Liu *et al*.^40^ presented results for the urban site of HFE giving a median value of 1.7 ± 0.2 for PM_10_, close to our value of 1.6 ± 0.3. The longest time series corresponds to the rural polluted site of SGP. Jefferson *et al*.^57^ summarized these measurements and gave mean values for PM_10_ and PM_1_ of 1.8 ± 0.4 and 1.9 ± 0.4, respectively. An earlier study at SGP^22^ found median *f*(RH = 85%/RH = 40%) values of 1.8 and 1.9 for PM_10_ and PM_1_ respectively, while in our study we obtain median values of 2.0 and 2.1(±0.6) for the same size cuts. For KCO, Clarke *et al*.^68^ gave the fit parameters for Eq. 2 and PM_1_ measurements. The retrieved mean value for *f*(RH = 85%/RH = 40%) using those parameters is 1.7 and the value obtained with the analysed data of this study is also 1.7 ± 0.1.

At PVC, Titos *et al*.^29^ reported mean values of *f*(RH = 80%/RH_dry_) for the whole campaign segregated by PM_10_ and PM_1_. They found values of 1.9 ± 0.3 for PM_10_ and 1.8 ± 0.4 for PM_1_, while in our study these values are: 1.9 ± 0.4 and 2.0 ± 0.6, respectively. For FKB, a range of *f*(RH = 80%/RH_dry_) values between 1.1 and 1.5 was given by Fierz-Schmidhauser *et al*.^23^. Our results show 25 and 75 percentile values of 1.3 and 1.7, with a mean value of 1.6 ± 0.3.

For FIK, the value of *f*(RH = 80%/RH_dry_) obtained in our study is 2.2 ± 0.2 is significantly lower than values of *f*(RH = 80%/RH_dry_) obtained by Stock *et al*.^69^ which ranged between 2.7–3.5. This discrepancy is likely due to (a) different methodology for obtaining *f*(RH) and (b) significant differences in measurement periods for the two studies. While our study used measurements of particle light scattering at dry and wet conditions to determine the enhancement factor, Stock *et al*.^69^ simulated the value of scattering at ambient conditions by means of an optical model using measurements of dry scattering and particle number size distribution and estimates of the complex index of refractive (derived from combining measured dry and wet size distributions and optical measurements) and ambient RH as input parameters. Additionally, our value represents the mean *f*(RH = 80%/RH_dry_) for one year of measurements, while Stock *et al*.^69^ only considered a three-day period with clean marine air masses.

To further assess the quality of our dataset, quality checks can be carried out in the form of closure studies at sites where the required additional measurements (chemical composition and aerosol size distribution) are available. This has been done previously for most PSI sites. For example, Fierz-Schmidhauser *et al*.^52^ compared the measured *f*(RH) at MHD with the corresponding value simulated by Mie theory (using the measured aerosol size distribution and the complex refractive index determined from chemical composition as inputs). Zieger *et al*.^51^ also performed closure studies at MEL to compare the measured and calculated dry scattering coefficient and the scattering enhancement factor. Similar closure studies were also done for HYY^26^, JFJ^46^ (for a different time period) and CES^12^.

Another type of closure study that could be used to assess these data is to determine if surface scattering coefficients, adjusted to ambient RH are consistent with remotely sensed vertical profile data, as was done in Zieger *et al*.^13^, where nephelometer measurements of aerosol hygroscopicity were compared to lidar and MAX-DOAS observations.

## Usage Notes

Researchers using this database can decide to use *Level 0* or *Level 1* light scattering coefficients measurements, the interpolated light scattering coefficients at a given RH, or the calculated scattering enhancement factors: *f*(RH_wet_/RH_dry_) and *f*(RH_wet_/RH = 40%) (*Level 2*). All these quantities are given for total and back scattering, but for different wavelengths depending on the site (see Table 2).

*Level 0* light scattering coefficients may be useful if a user wants to carry out their own data processing and corrections. Light scattering coefficients from *Level 1* may be useful if a user wants to perform their own fit and try a different equation than the used in this study (Eq. 2). The user can also calculate their own *f*(RH = 85%/RH_dry_) or *f*(RH = 85%/RH = 40%) and compare to our findings. This dataset can be useful to perform closure studies, light scattering coefficients can be compared to the outputs from Mie calculations obtained using size distribution or chemical data.

The entire dataset and metadata can be accessed via the ACTRIS Data Centre^67^, while the data for the individual sites can be accessed via the EBAS Data Centre (http://ebas.nilu.no/) which also includes further online visualization tools and other site specific services such as further atmospheric observational data and air mass trajectory calculations. The data files are provided in the NASA Ames format and EBAS providesa useful Python package for accessing and reading the files (see repository at https://git.nilu.no/ebas/ebas-io). Further reading files (e.g. for Matlab) are provided by the authors upon request.

## ISA-Tab metadata file

Download metadata file

## Data Availability

The Matlab code used to generate this dataset is available from the corresponding authors upon request. The repository contains 26 different scripts due to the site dependent characteristics. These scripts read the WetNeph and DryNeph raw files as well as the *T*/RH sensor files. The sample RH is established for each particular case as explained in this text, and flags for size cut and valid/invalid measurements are also set taking into consideration each particular case. The user can find detailed explanations of the various corrections applied to the raw measurements in the literature as cited in this paper. The codes apply the corrections to the raw measurements, obtaining the resulting corrected quantities for *σ*_sp_(RH_dry_), *σ*_bsp_(RH_dry_), *σ*_sp_(RH_wet_) and *σ*_bsp_(RH_wet_). The user can use this dataset to apply their own methodology for obtaining *f*(RH) and/or applying empirical regressions to the measured humidograms.
